# Actinomycetes from Sediments in the Trondheim Fjord, Norway:  Diversity and Biological Activity

**Published:** 2008-01-23

**Authors:** Harald Bredholt, Espen Fjærvik, Geir Johnsen, Sergey B. Zotchev

**Affiliations:** 1 Alpharma AS, Harbitzaleen 3, 0275 Oslo, Norway; 2 Department of Biotechnology, Norwegian University of Science and Technology, 7491 Trondheim, Norway; 3 Department of Biology, Norwegian University of Science and Technology, 7491 Trondheim, Norway

**Keywords:** Actinomycete bacteria, fjord sediments, molecular taxonomy, antimicrobial activities

## Abstract

The marine environment represents a largely untapped source for isolation of new microorganisms with potential to produce biologically active secondary metabolites. Among such microorganisms, Gram-positive actinomycete bacteria are of special interest, since they are known to produce chemically diverse compounds with a wide range of biological activities. We have set out to isolate and characterize actinomycete bacteria from the sediments in one of the largest Norwegian fjords, the Trondheim fjord, with respect to diversity and antibiotic-producing potential. Approximately 3,200 actinomycete bacteria were isolated using four different agar media from the sediment samples collected at different locations and depths (4.5 to 450 m). Grouping of the isolates first according to the morphology followed by characterization of isolates chosen as group representatives by molecular taxonomy revealed that *Micromonospora* was the dominating actinomycete genus isolated from the sediments. The deep water sediments contained a higher relative amount of *Micromonospora *compared to the shallow water samples. Nine percent of the isolates clearly required sea water for normal growth, suggesting that these strains represent obligate marine organisms. Extensive screening of the extracts from all collected isolates for antibacterial and antifungal activities revealed strong antibiotic-producing potential among them. The latter implies that actinomycetes from marine sediments in Norwegian fjords can be potential sources for the discovery of novel anti-infective agents.

## Introduction 

The demand for new antibiotics continues to grow due to the rapid spread of antibiotic-resistant pathogens causing life-threatening infections. Although considerable progress is being made within the fields of chemical synthesis and engineered biosynthesis of antimicrobial compounds, nature still remains the richest and the most versatile source for new antibiotics [1,2,3]. 

Bacteria belonging to the family *Actinomycetaceae* are well known for their ability to produce secondary metabolites, many of which are active against pathogenic microorganisms. Traditionally, these bacteria have been isolated from terrestrial sources although the first report of mycelium-forming actinomycetes being recovered from marine sediments appeared several decades ago [4]. It is only more recently that marine-derived actinomycetes have become recognized as a source of novel antibiotics and anti-cancer agents with unusual structures and properties [5]. 

Many microbiologists believe that free-living bacteria are cosmopolitan due to their easy dispersal [6]. However, chemical and physical factors contribute to selection of species and strains that are best adapted to that particular environment. Due to the broad bacterial species definition one may find members of one species in two very different environments [7,8]. However, comprehensive analysis of the recent studies strongly suggests that free-living microbial taxa exhibit biogeographic patterns [7,9]. Some of the unusual structures and properties of compounds isolated from marine sources and the fact that 58 % of the isolated actinomycetes from sediments collected around Guam in the Pacific ocean required sea water for growth [5] implies that one may find microorganisms adapted to the marine environment and producing compounds not found among microorganisms adapted to the terrestrial sources. 

Fjords are narrow inlets of the sea, which have been formed as a result of marine inundation of glaciated valleys. Typical characteristics of a fjord include a relatively narrow inlet, significantly eroded bottom and communication with the open sea. The ecology of the microorganisms, especially bacteria, inhabiting the fjords is poorly studied. The Trondheim fjord (135 km long) differs from many other fjords by a large fresh water supplement from six major river systems. In one year these rivers bring fresh water to the fjord corresponding to 6.5 % of it total water volume [10]. From May until September the fjord contains a 5-25m thick layer of brackish surface water, depending on time, weather and location. In the brackish water layer the salt content varies from approximately 18 to 32 practical salinity units (PSU). The dissolved and particulate organic matter in the sediments from the fjord has both marine origin from phytoplankton and macroalgae as well as terrestrial from soil due to fresh water run-off, highly influenced by snow melting in April-May.

The Norwegian marine environments are largely unexplored, and may provide a rich source of the microorganisms producing novel and efficient anti-infective compounds. The purpose of this study was to investigate if fjord sediments from temperate areas could be suitable sources for isolation of mycelium-forming actinomycetes producing antimicrobial compounds. 

## Results  

### Collection and analysis of the sediment samples

Sediment samples from two different locations and five depths were collected and processed (see Table 1 for description and ID). The first site (B-site) was close to the shore and here samples were collected at depths of 4.5, 6, 27 and 28 m. The 4.5 m and 6 m sampling sites were situated in the kelp forest belt and the 6 m sampling site had a brown layer of sedimented micro algae on the surface.  At 27 and 28 m depth, the sediment surface was dominated by sand/silt. The second site (T-site) was located in the middle of the fjord and here samples were taken at 450 m depth, where the sediment was dominated by clay particles. Visual inspection indicated a higher content of fine organic matter than in the other sediments. Similar to the B2 sample the sediments from this location also had a layer of dead micro algae on the surface.

**Table 1:** Description of sediments and sampling sites.

Site	Sample ID	Depth (m)	Sediment type	Vegetation	Org C(%)	Org N(%)
Biologen	B1	4.5	Fine mud with small stones	+	0.7	0.04
Biologen	B2	6.0	Clay and stones	++	1.1	0.06
Biologen	B3	27	Fine muddy sand	–	0.6	0.05
Biologen	B4	28	Clay	–	0.7	0.04
Trollet	T1	450	Clay with fine organic material	–	1.8	0.15

The contents of organic material in the different sediment samples were measured as total carbon (C) and nitrogen (N), in order to test possible correlation with actinomycete microbiota and the ability to produce antimicrobial compounds. The highest content of organic carbon (1.8 %) and nitrogen (0.15 %) was found in the T1 sediment (Table 1). The contents of organic carbon and nitrogen in the B-site sediments varied from 0.6 to 1.1 % and 0.04 to 0.06 %, respectively, where the B2 sample was clearly the one with highest content of the organic matter. Higher total N over C ratio may indicate a higher content of more rapidly degradable organic material [11]. The highest N/C ratio was found for the B3 and T1 sediments, suggesting the presence of more easily degradable organic matter in these samples.

### Isolation of actinomycete bacteria

Settled suspensions of the sediments were plated onto four different selective media adding up to a total of 320 primary isolation plates (diameter 14 cm) of which 248 (78%) yielded mycelium-forming actinomycete colonies. The total number of actinomycetes observed on the primary isolation plates was 7874. An average of 24.6 mycelium-forming actinomycete colonies per plate were observed with numbers increasing to 31.8 when only considering those plates that yielded actinomycetes. 3200 colonies were picked based on actinomycete-like morphology, of which around 900 formed powdery colonies with well-developed aerial hyphae fragmented into spore chains. These isolates were tentatively termed as *Streptomyces*-like actinomycetes*. *The main part of the remaining isolates formed orange to red pigmented colonies with solid colony texture, non fragmenting substrate mycelium that lacked aerial hyphae and often turned purple, brown or black upon sporulation, and was tentatively termed as MNSA (mycelium-forming non-streptomycete actinomycetes)*.* The total CFU (colony forming units) for *Streptomyces*-like and MNSA isolates were registered (Table 2). The highest total CFU was found on soil agar from the B1 sample and the highest number of MNSA colonies was found in the same sediment when plated onto chitin agar (IM7b). Highest number of *Streptomyces*-like colonies was found when Biologen 6 m sediments were plated on to IM6 (modified Kusters) agar.

**Table 2:** Viable counts of bacteria and actinomycetes in sediments from different depths in the Trondheim fjord after plating onto selective agar media. The numbers are mean values of CFU (colony forming units) per mL wet sediment.

Sample ID	Medium	CFU/mL wet sediment		
		Bacteria	*Streptomyces*-like	Non-*Streptomyces* actinomycetes
B1	IM5b	1.2 x 10^6^	2.5 x 10^2^	5.0 x 10^2^
	IM6b	9.5 x 10^4^	5.5 x 10^2^	2.0 x 10^3^
	IM7b	8.4 x 10^4^	1.5 x 10^3^	2.4 x 10^4^
	IM8b	8.0 x 10^4^	2.0 x 10^3^	1.8 x 10^4^
B2	IM5b	5.4 x 10^5^	2.5 x 10^3^	5.0 x 10^3^
	IM6b	8.1 x 10^4^	6.0 x 10^3^	1.5 x 10^3^
	IM7b	7.4 x 10^4^	2.0 x 10^3^	1.6 x 10^4^
	IM8b	1.1 x 10^5^	2.3 x 10^3^	1.6 x 10^4^
B3	IM5b	5.1 x 10^5^	1.5 x 10^2^	N.D
	IM6b	1.0 x 10^5^	2.0 x 10^2^	3.5 x 10^3^
	IM7b	7.7 x 10^4^	2.0 x 10^2^	1.3 x 10^4^
	IM8b	2.6 x10^5^	5.0 x 10^2^	1.1 x 10^4^
B4	IM5b	3.6 x 10^5^	5.0 x 10^1^	5.0 x 10^2^
	IM6b	1.2 x 10^5^	4.0 x 10^2^	3.5 x 10^2^
	IM7b	9.4 x 10^4^	7.0 x 10^2^	1.6 x 10^4^
	IM8b	1.4 x 10^5^	9.0 x 10^2^	1.7 x 10^4^
T1	IM5b	2.5 x 10^4^	N.D.	1.3 x 10^3^
	IM6b	7.2 x 10^3^	7.5 x 10^1^	6.7 x 10^3^
	IM7b	8.0 x 10^3^	N.D.	2.3 x 10^3^
	IM8b	6.3 x 10^3^	2.5 x 10^1^	2.5 x 10^3^
N.D. - Not detected, no colonies on the agar plates. Plates were incubated at 20 ^o^C for up to 4 weeks.				

The T1 sample contained the highest relative number of MNSA colonies (93 %) and the lowest number of *Streptomyces*-like colonies (0.7 %) (Table 2). However the total CFU on selective media from this sediment was approximately one tenth of that from the B-site sediments. For the latter sediments the highest relative numbers of MNSA colonies varied from 17 to 30 % when plated on to IM7b agar. In general, the IM7b (colloid chitin) gave the highest numbers of MNSA colonies with the exception of the T1 sample where IM6 was clearly the best in this respect. IM5 (humic acid sea water agar) was the isolation medium that gave the lowest number of mycelium-forming actinomycetes from fjord sediments.

**Figure 1:**
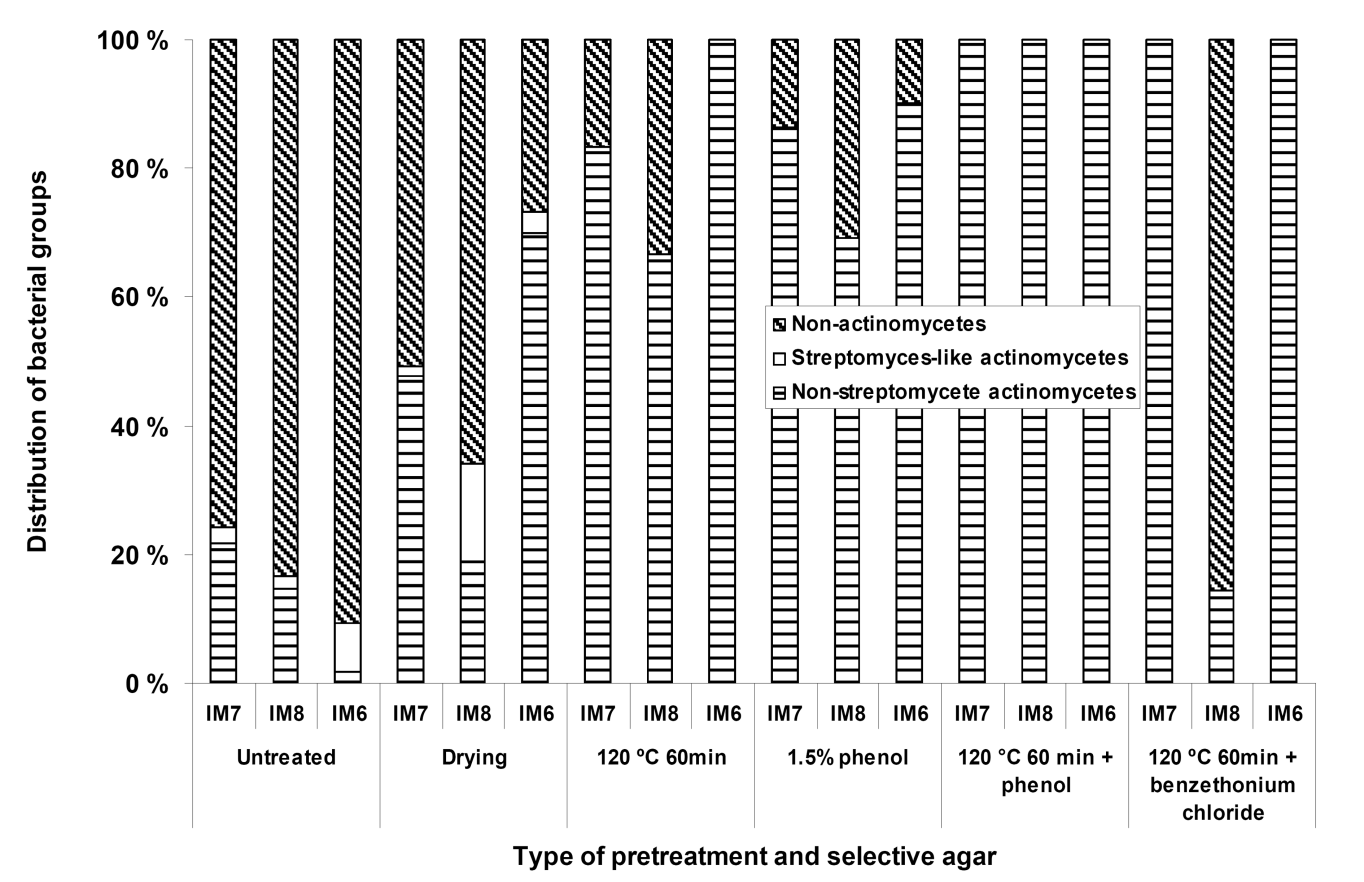
The effect of different types of pre-treatments, applied to the sediments samples, on the relative numbers of actinomycetes appearing on the isolation plates

Different types of selective pre-treatments were applied in order to increase the number of mycelium-forming actinomycetes relative to the non-actinomycetal heterotrophic microbial flora. These treatments included dry heat, phenol treatment, dry heat followed by phenol treatment, dry heat followed by benzethonium chloride treatment, and pollen baiting. For the B-site sediment samples (Figure 1) it was possible to obtain increased relative numbers of actinomycetes on the agar plates with all the different types of pretreatments, except for the pollen baiting, which did not yield any isolates. With some of the pretreatments (dry heat followed by phenol treatment), it was possible to obtain isolation plates only containing actinomycetes with morphologies typical for the genera *Micromonospora*.

However, for the T1 (450 m) samples these pretreatments were detrimental, and apparently eliminated most of the actinomycete microbiota. We were not able to isolate any mycelium-forming actinomycetes from the sediments with the pollen baiting technique, suggesting that actinomycetes producing zoospores are rare in the fjord sediments we have investigated. 

### Sea water requirement

All original media for isolation of actinomycetes contained sea water, and we decided to test whether the presence of this media component can be crucial for growth of isolated actinomycetes. Therefore, the isolates were transferred to the respective media with or without sea water, and their growth was monitored over a period of 8 weeks. Growth of approximately 8 % percent of the isolates from T1 sample was found to be completely dependent on the presence of sea water, while the respective average figure for the B-site samples was 9 %. The individual percentages for the B-site samples were: 4.5 m; 8 %; 6 m; 10 %, 27 m; 5 % and 28 m; 8 %. In addition, around 20 % of the isolates grew considerably faster on agar media containing sea water. The major part of the isolates (50 %) did not show any clear preference for media with or without sea water, while around 20 % of the isolates grew better in the absence of sea water. 

### Biological activities

**Figure 2:**
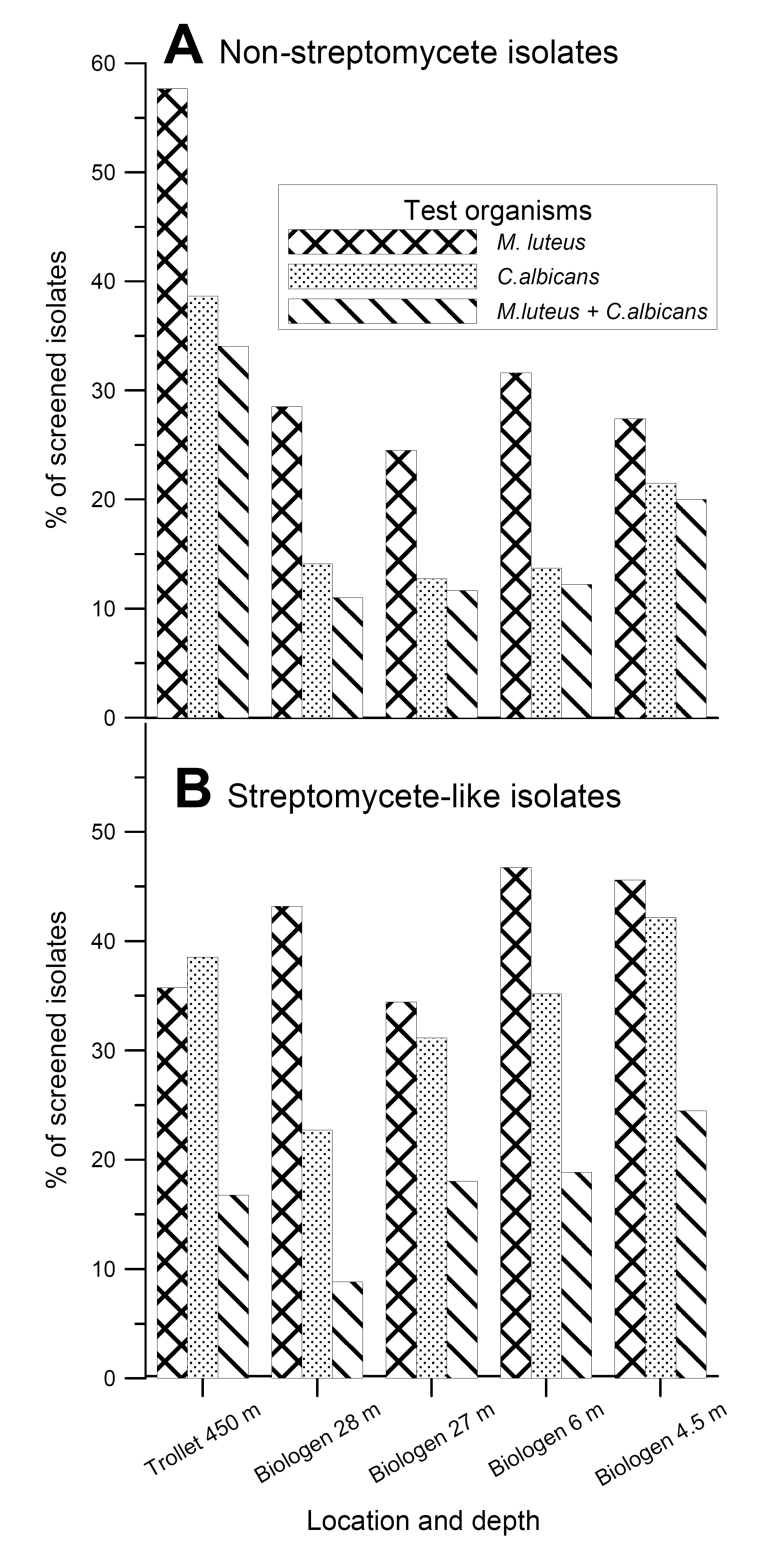
The****percentage of the mycelium-forming actinomycete isolates displaying antibacterial activities against *Micrococcus luteus *and antifungal activities against *Candida albicans* in agar diffusion assays. **A**. Non-streptomycete isolates. **B**. *Streptomyces*-like isolates.

The 3,200 isolated actinomycete bacteria were transferred to three different solid (agar) production media. After an incubation period of one to six weeks depending on growth rate of the isolates, the media and the cells were dried and extracted with DMSO. The extracts were tested for antibacterial and antifungal activity against the Gram-positive bacterium *Micrococcus luteus *and the yeast* Candida albicans *using traditional agar diffusion assays*.* The results of this analysis are presented in Figure 2. 

Somewhat surprisingly, the MNSA isolates from the T1 sediments showed the highest frequency of activity against Gram-positive bacterium *M. luteus* (58 %) and yeast *C. albicans* (39 %). For the B-site MNSA isolates the frequency of the corresponding activities varied between 25-32 % and 13-21 %, respectively. The frequency of activities against *M. luteus* and *C. albicans* among the *Streptomyces*-like strains varied between 34-47 % and 23-42 %, respectively. Within this group, the highest frequency of antibacterial activities was found among the isolates from the B2 sample and the highest antifungal activity among the B1 sample. 

### Molecular taxonomy

**Figure 3:**
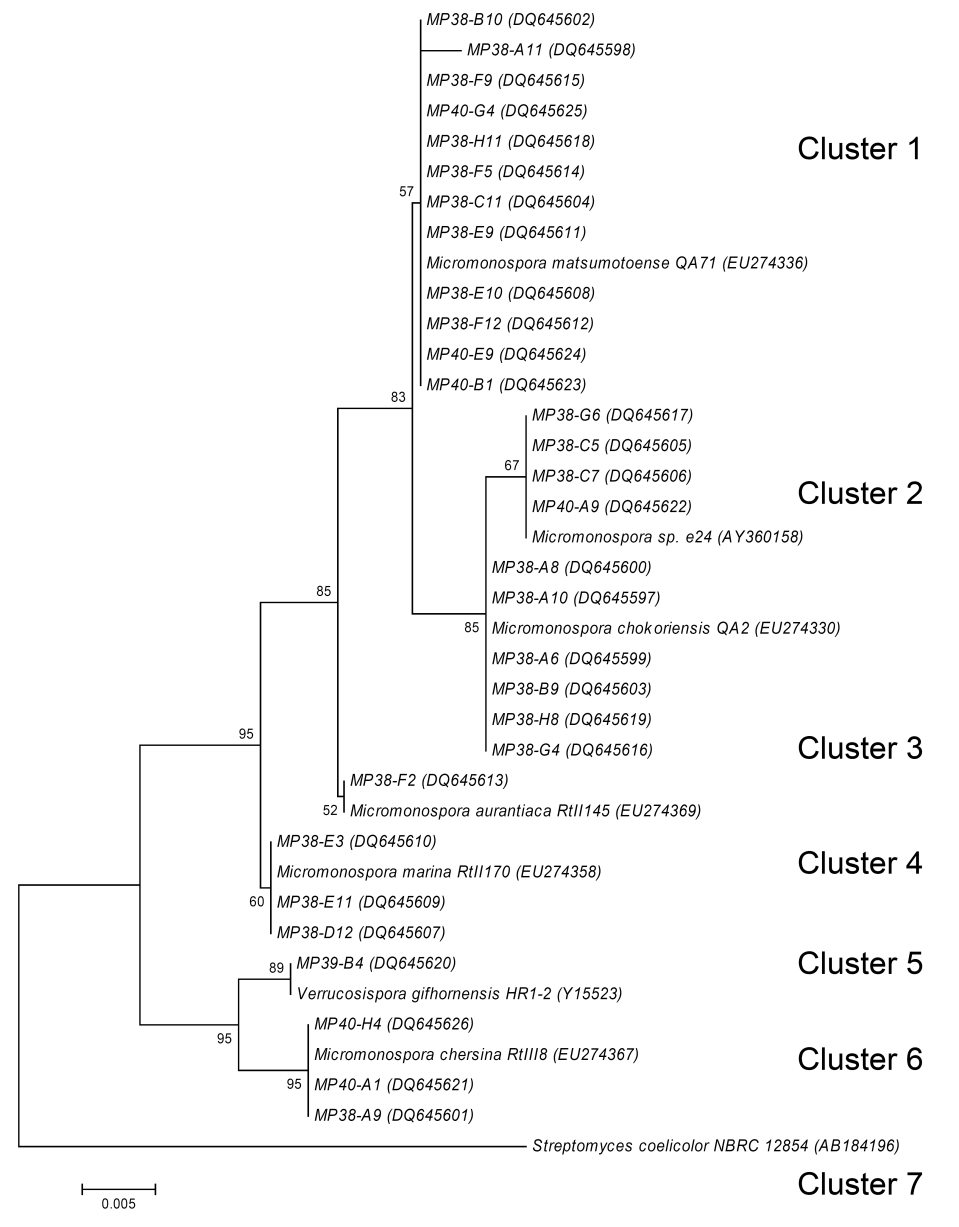
Phylogenetic relationship of partial 16S rDNA sequences generated in this study, rooted using the 16S rDNA sequence of *Streptomyces coelicolor*. See Material and methods for tree description. Numbers at tree nodes represent the number of times the topology to the right of the node was recovered in 100 bootstrap re-samplings; values lower than 50 are not shown. Accession numbers for the sequences are in parentheses. Scale bar represents the number of changes per base position.

Examination of the colony morphology of the actinomycete isolates suggested that they may tentatively belong to the genera *Streptomyces* and *Micromonospora*. In order to confirm preliminary classification, we selected 30 isolates from the T1 sample having different “representative” morphologies. These isolates were morphologically more homogeneous than the B-site isolates. Fragments of the 16S rRNA genes from these isolates were PCR-amplified, sequenced, and a phylogenetic tree was constructed, allowing the sorting of the sequences into seven different clusters (Figure 3). Cluster 1 consisted of 12 isolates showing 99 % to 100 % homology to *Micromonospora matsumotoense.* All displayed moderate antibacterial activity, except MP38-F9 and MP38-F5 who displayed strong and no activity respectively. They all also produced a weak antifungal activity, except MP38-F5. Cluster 2 contained four isolates showing 99 to 100 % to *Micromonospora sp. e24. *None of these isolates showed antibacterial or antifungal activity. ****The****six isolates in Cluster 3 showed 99 to 100 % identity to *M. chokoriensis*. Of these isolates only MP38-A6 showed antimicrobial activity which was moderate antibacterial and weak antifungal. Cluster 4 consisted of one isolate with its partial 16S rDNA sequence showing 99 % identity to *M. aurantiaca*. This isolate showed no antibacterial or antifungal activity. Cluster 5 contained three isolates that showed 99 % homology to *M. marina*. Two of the isolates MP38-E11 and MP38-D12 displayed moderat antibacterial activity. Cluster 6 consisted of one isolate showing 100 % homology to *Verrucosispora gifhornensis.* No antimicrobial activity was detected for this strain. Cluster 7 contained three isolates which showed 99 % homology to *M. chersina*, all of them showing antibacterial activity. Despite clear division of the isolates to different clusters according to the partial 16S rDNA sequences, there was just as much morphological variation among the isolates within the clusters as between the different clusters. For example, among 12 *Micromonospora* isolates from Cluster 1, only three have shown differences in the partial 16S rDNA sequences However, all isolates in this cluster exhibited differences in morphology, and some of them were clearly different in terms of biological activity profiles.

## Discussion 

The Trondheim fjord is approximately 135 km long and is characterized by the large water supply from six rivers entering the fjord. The dissolved and particulate organic matter in the sediments from the fjord originates from both marine phytoplankton and macro algae, and terrestrial material from the run-off caused by snow melting in the spring brought to the fjord by the rivers. An average of 9 % of the actinomycete isolates in this investigation required sea water for growth, most of them originating from the deep-water sediment sample. The latter can probably be explained by a better adaptation of the bacteria in this sediment to the sea water environment, since fresh water supply at such depth shall be minimal. Jensen *et al*. [5] reported that as much as 58 % of the actinomycetes, isolated from sediment samples collected around the island of Guam required sea water for growth. While the Trondheim fjord is a sea inlet into the terrestrial environment, Guam is positioned far out into the Pacific Ocean. These facts suggests that although there is a clear evidence of metabolically active marine actinomycetes in the fjord sediments, there is also a considerable number of actinomycetes of terrestrial origin present as well. 

*Micromonospora* and *Streptomyces*-like actinomycetes were the dominating actinomycetes isolated from the near shore shallow water sediments, where the numbers of streptomycetes decreased with depth and distance from the shore, as have also been reported by others [12]. However, a considerable number of isolates displayed morphologies that did not conform with these two genera. Their relative phylogenetic positions are under investigation and will be reported elsewhere.

The use of different types of selective treatments originally designed for selective isolation of actinomycetes from soil increased the relative numbers of actinomycetes on the agar plates inoculated from the shallow water (4.5 to 28 m) near shore samples. Although these techniques have previously been applied with success for isolation of groups of actinomycetes from soil [13-15], no reports exist to our knowledge on effectiveness of such techniques applied to the marine sediments from temperate areas. For the deep-water sediment samples (450 m) these treatments were clearly detrimental, mainly resulting in agar plates free for actinomycetes. This suggests that the selective pre treatments designed to isolate actinomycetes from soil are not optimal for marine samples and more effort is required in order to establish methods allowing specific enrichment of marine actinomycetes. The dominating actinomycete genus isolated from the deep sediments under the conditions tested was *Micromonospora*. Some of the selective treatments as for example dry heat at 120 °C for 60 min and exposure to 1.5 % phenol is indeed designed for the selective isolation of *Micromonospora*. However, very few *Micromonospora* isolates from the deep water samples survived these treatments. This could suggest that although their relative phylogenetic position determined by 16S rDNA is identical or close to known terrestrial organisms, they still have signs of adaptation to their marine environment. *Micromonospora* species growing at 450 m experience an environment with relatively low and constant ambient temperature (8 °C), stable and high salinity, and are not exposed to desiccation and may therefore differ significantly from their terrestrial counterparts. Without the use of selective pre-treatments the relative numbers of actinomycetes (mainly *Micromonospora*) could account for as much as 90 % of the colonies on some of the selective agar media. At the moment, however, we can not exclude the possibility that the dominance of *Micromonospora* on our isolation plates was caused by the chosen isolation procedures, and that other actinomycetes are also present in considerable numbers in these sediments. 

Although we have analyzed too few samples to draw any solid conclusions, the trend in our results was that the percentage of isolates displaying activity against *M. luteus* among the MNSA isolates was roughly proportional to the carbon content in the different sediments. That is, the percentage of mycelium-forming actinomycetes with this activity increased with the content of organic carbon in the sediments (Table 1 and Figure 2). No correlation could be seen between sampling depth and antimicrobial activity among MNSA isolates. With the exception of the T1 sample (collected from 450 m depth), there was a decrease in the percentage of *Streptomyces*-like isolates displaying activity against *C. albicans* with increasing sampling depth. At the same time, we are aware of the fact that our test organism, *Candida albicans*, is of terrestrial origin, and thus we might have missed antibiotic activities directed against marine fungi.

Despite the fact that the T1 sample had the highest content of organic carbon and nitrogen, it gave the lowest CFU number on our selective media. It also contained less morphological diversity among the actinomycete isolates than what was found for the shallow water B-site samples. The reason for this is most probably our failure to cultivate many of the actinomycetes present in the sample due to their need for special cultivation conditions. This is supported by previous reports where high actinobacterial diversity was found in marine sediments by constructing actinobacterium-specific 16S rDNA clone libraries. Furthermore, the information from the cultivation-independent techniques could be used to improve the recovery of novel actinobacteria [16-18]. Even though, the diversity and biological activities of actinomycetes from the Trondheim fjord sediments unraveled so far suggests that they might be a rich source for discovery of new anti-infective agents. 

## Experimental Section 

### Sample collection and isolation of bacteria

Sediments from 4.5, 6.0, 27 and 28 m depths were collected by scuba divers, while sediments from 450 m depth were sampled with a box-corer. The upper 5 cm of the sediments were collected in zip-lock bags (scuba) or with a sterile spade (box –corer) and transferred to 1 liter sterile plastic containers. Approximately 10 % of the container volume was filled with 60 % sediment and 40 % sea water from the sampling site. This was done in order to ensure aerobic conditions under storage upon processing. Samples were processed the same day or the day after sampling. All storage was done in the dark at 4 ºC. Sediments were diluted 1:10 v/v with sterile sea water and vigorously shaken with glass beads for 30 sec. Settled (5 min) sediment suspensions were plated on to different selective agar media and incubated at 20 °C for two to six weeks. Selective treatments were performed on dried sediments (Speedvac 30 °C, 16 h) and included dry heat (120 °C, 60 min), phenol (1.5 %, 30 min at 30 °C), dry heat and phenol, dry heat and benzethonium chloride (0.02 %, 30 min at 30 °C), as well as pollen baiting [19,20].

### Carbon and nitrogen content analysis 

Sediment samples were dried and ground with a pester and mortar, before the total carbon (C) and nitrogen (N) content in the sediment samples were measured using a NA 1500 Nitrogen/Carbon/Sulphur analyzer from Carlo Erba Instruments. To ensure good average values each sediment sample was analyzed in five parallels.

### Isolation and production media 

Isolation media consisted of the following: IM5 (humic acid agar [21], with sea water), humic acid (1 g), K_2_HPO_4_ (0.5 g), FeSO_4_∙7H_2_O (1 mg), agar (20 g), vitamin B solution (1 mL), natural sea water (0.5 L) and distilled water (0.5 L); IM6 glycerol (0.5 g), starch (0.5 g), sodium propionate (0.5 g), KNO_3_ (0.1 g), asparagine (0.1 g), casein (0.3 g), K_2_HPO_4_ (0.5 g), FeSO_4_∙7H_2_O (1 mg), agar (20 g), vitamin B solution (1 mL), natural sea water (0.5 L) and distilled water (0.5 L); IM7 (chitin agar [9], with sea water) chitin (Sigma), K_2_HPO_4_ (0.5 g), FeSO_4_∙7H_2_O (1 mg), agar (20 g), vitamin B solution (1 mL), natural sea water (0.7 L) and distilled water (0.3 L); IM8, malt extract (1 g), glycerol (1 g), glucose (1 g), peptone (1 g), yeast extract (1 g), agar (20 g), natural sea water (0.5 L) and distilled water (0.5 L). The pH of the isolation media was adjusted to pH 8.2. Vitamin B solution consisted of the following: thiamine-HCl (50 mg), riboflavin (50 mg), niacin (50 mg), pyridoxine-HCl (50 mg), inositol (50 mg), Ca-pantothenate (50 mg), *p*-aminobenzoic acid (50 mg), biotin (25 mg) and distilled water (100 mL). All isolation media were amended with filtered (0.2-µm pore size) cycloheximide (50 µg/mL), nystatin (75 µg/mL) and nalidixic acid (30 µg/mL).  

Production media: PM2, mannitol (5.0 g), soya bean flour (5.0 g), Clerol (antifoam, 0.1 g), dry yeast (0.9 g), agarose (10.0 g), tap water (1 L); PM3, oatmeal (20 g), glycerol (2.5 g), FeSO_4_∙7H_2_O (0.1 mg), MnCl_2_∙4H_2_O (0.1 mg), ZnSO_4_∙7H_2_O (0.1 mg), agarose (10 g), tap water (1 L); PM4, glucose (0.5 g), glycerol (2.5 g), oatmeal (5.0 g), soybean meal (5.0 g), yeast extract (0.5 g), casaminoacids (2.0 g), CaCO_3 _(2.0 g), Clerol (0.1 g), agarose (10 g) and tap water (1 L).

Media for nucleic acid extraction: organic agar Gause 2 (modified), glucose (10 g), trypton (3 g), peptone (5 g), agar (20 g), tap water (0.5 L) and sea water (0.5 L).

Plates with isolation and production media were incubated at 20 ^o^C for periods of 2 to 6 weeks.

### Cultivation, extraction and bioactivity testing

The 3200 isolated mycelium-forming actinomycetes were transferred to three different solid (agar) production media PM2, PM3 and PM4 in 96-well plates. After an incubation at 20 °C for one to six weeks, depending on growth rate of the isolates, the media and the cells were dried directly in the plates, and extracted with dimethylsulfoxide (DMSO, Sigma, 200 mL). The extracts were tested for antibacterial and antifungal activity against the Gram-positive bacterium *Micrococcus luteus* ATCC 9341 and the yeast *Candida albicans* ATCC 10231 using traditional agar diffusion assays. Each DMSO extract (1 µL) was applied onto the surface of the agar inoculated with the test organism and activity registered as inhibition zones after 16 hours of incubation at 34 °C. 

### Nucleic acid extraction, 16S rDNA amplification, sequencing and analysis 

Fresh colonies grown on Gause 2 organic agar [22] were macerated and transferred to sterile distilled water (100 μL) and heated to 98 °C for 10 min. The suspensions were centrifuged (5000 x g, 1 min) and DNA from the clear supernatant precipitated with three volumes of ethanol, centrifuged (12000 rpm, 15 min) and pellets dissolved in distilled water to the original volume. 

16S ribosomal DNA (rDNA) sequencing templates were amplified from genomic DNA by PCR using previously described [18] actinomycete specific primers S-C-Act-235-S-20 (5’-CGCGGCCTATCAGCTTGTTG-3’) and S-C-Act-878-A-19 (5’-CCGTACTCCCCAGGCGGGG-3’). Reaction mixture (50 µL) contained genomic DNA extract (1 µL), Thermopol Buffer (New England Biolabs), DMSO (2 µL), Bovine serum albumin (2 µL), deoxynucleoside triphosphate mixture (2.5 pmol), each primer (20 pmol), and Taq DNA polymerase (2.5 U). All sequencing reactions were carried out with an ABI PRISM 3100 genetic analyzer at the Department of Biology, The Norwegian University of Science and Technology. DNA sequences were deposited to GenBank under accession numbers DQ645597 to DQ645626. The 16S rDNA sequences (500-625 bp) were used to search the GeneBank database with the BlastN algorithm to reveal closest matches to the 16S rDNA sequences for known species. Sequences were aligned with representative actinomycete 16S rDNA sequences and a phylogenetic tree was constructed using the Molecular Evolutionary Genetics Analysis (MEGA) software version 4.0 [23].
